# *Fraxinus*: A Plant with Versatile Pharmacological and Biological Activities

**DOI:** 10.1155/2017/4269868

**Published:** 2017-11-27

**Authors:** Iqra Sarfraz, Azhar Rasul, Farhat Jabeen, Tahira Younis, Muhammad Kashif Zahoor, Muhammad Arshad, Muhammad Ali

**Affiliations:** ^1^Department of Zoology, Faculty of Life Sciences, Government College University Faisalabad, Punjab 38000, Pakistan; ^2^Department of Zoology, Faculty of Life Sciences, University of Sargodha, Punjab 40100, Pakistan

## Abstract

*Fraxinus*, a member of the Oleaceae family, commonly known as ash tree is found in northeast Asia, north America, east and western France, China, northern areas of Pakistan, India, and Afghanistan. Chemical constituents of* Fraxinus* plant include various secoiridoids, phenylethanoids, flavonoids, coumarins, and lignans; therefore, it is considered as a plant with versatile biological and pharmacological activities. Its tremendous range of pharmacotherapeutic properties has been well documented including anticancer, anti-inflammatory, antioxidant, antimicrobial, and neuroprotective. In addition, its bioactive phytochemicals and secondary metabolites can be effectively used in cosmetic industry and as a competent antiaging agent.* Fraxinus* presents pharmacological effectiveness by targeting the novel targets in several pathological conditions, which provide a spacious therapeutic time window. Our aim is to update the scientific research community with recent endeavors with specifically highlighting the mechanism of action in different diseases. This potentially efficacious pharmacological drug candidate should be used for new drug discovery in future. This review suggests that this plant has extremely important medicinal utilization but further supporting studies and scientific experimentations are mandatory to determine its specific intracellular targets and site of action to completely figure out its pharmacological applications.

## 1. Introduction

Plant derived natural products have been a continuous source of inspiration for human therapeutics despite enormous pharmaceutical industries relying on synthetic chemistry methods for drug discovery [1].

Over the centuries, natural products from plants have proven their worth as a primary source of novel chemical entities having therapeutic potential [2]. Natural products being enriched with variety of anticancer, antioxidant, and neuroprotective compounds have a great potential for drug discovery [3]. Currently, drug discovery from plants is a multidimensional research approach including botanical, phytochemical, molecular, and biological techniques providing important and new leads against pharmacological targets in various pathological conditions [4]. The first record of sophisticated medicinal system from natural products dates back to 2600 BC in Mesopotamia, consisting of about 1000 medicines derived from botanical compounds and plant derivatives [5]. From 1981–2014, out of the 1562 drugs being approved, 1211 were small molecule approved drugs that are new chemical entities, which are nonsynthetic and obtained from natural products. Among them, 49% of anticancer drugs belong to natural products [6, 7]. In accordance with another record from 1981 to 2010, 1073 new drugs being approved, only 36% were synthetic entities and more than 50% were inspired or derived from nature [5]. About 80% of the population globally depends upon the botanical drugs mainly from medicinal plants [8].

Various studies conducted by researchers have reported that natural products have anticancer [9, 10], antibacterial [11], antifungal [12], antiviral [13], antioxidant [14, 15], anthelmintic [16], and anti-inflammatory [17, 18] activities. Particularly well-cited examples of natural products obtained from medicinal plants that have become prominent in modern pharmacotherapy as anticancer agents, include vinblastine and vincristine from* Catharanthus roseus* (Madagascar periwinkle), paclitaxel, and its derived compounds from Taxus species and camptothecin and its derivatives from* Camptotheca acuminata *Decne (Chinese tree) [5]. Cholinesterase inhibitor, galanthamine, as neuroprotective agent in Alzheimer's disease was firstly isolated from* Galanthus nivalis *L. [19]. Artemisinin, a potential antimalarial agent, was initially derived from Chinese medicinal herb* Artemisia annua *L. [20].

Traditionally, plant based drugs and medicinal plants have been employed for the treatment of humans and livestock in Pakistan and these medicinal plants play a valuable role in health care system in different areas of Pakistan [21].

*Fraxinus*, a member in the Oleaceae family, commonly known as ash tree [22] is found in various regions of world such as in western and eastern France [23], north American [24], north east Asia [25], China [26], north Pakistan, India, Afghanistan, Morocco, and Algeria. In northern areas of Pakistan, root bark and leaves of* Fraxinus* plant have been traditionally used for the cure of malaria and pneumonia [27]. A range of chemical constituents including secoiridoids, phenylethanoids, lignans, flavonoids, and coumarins has been isolated from* Fraxinus* plant. Metabolites and extracts from this plant have been found to possess variety of biological activities such as anticancer, anti-inflammatory, antioxidative, antimicrobial, hepatoprotective, antiallergic, skin regenerating, and diuretic [28, 29].

Esculetin, esculin, fraxin, and fraxetin are some of the pharmacologically active components isolated from different species of* Fraxinus* plant (Figure 2). Esculetin has been extensively used in Chinese herbal medicine due to its vast pharmacological activities such as antioxidant, anticancer, antibacterial, and anti-inflammatory [30].

This article is an effort to summarize the available information on pharmacological activities of different compounds isolated from traditional medicinal plant* Fraxinus* commonly known as ash tree. The searched literature highlights recent advances and scientific literature were checked through different sites, Elsevier ScienceDirect and PubMed, and relevant medical journals. The compiled data will hopefully update the scientific researchers with cotemporary endeavors in described field. We use “Fraxinus,” “Fraxinus and biological activity,” “medicinal plants,” “anti-inflammatory,” “anticancer,” and “anti-oxidant” as key words for search.

## 2. Biological Activities of* Fraxinus* Plant

**Figure 1:**
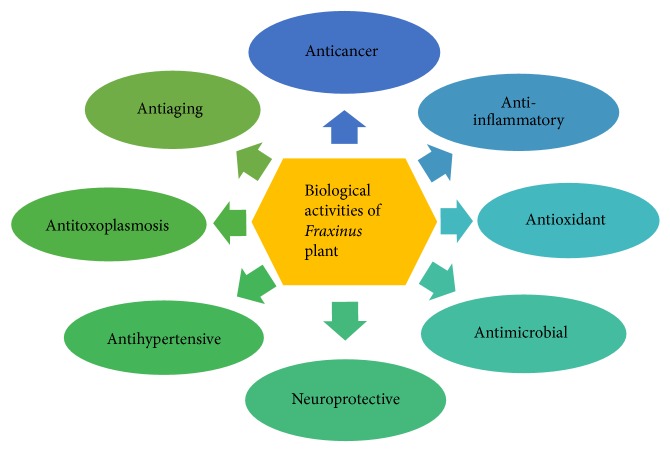
Biological activities of* Fraxinus* plant.

**Figure 2:**
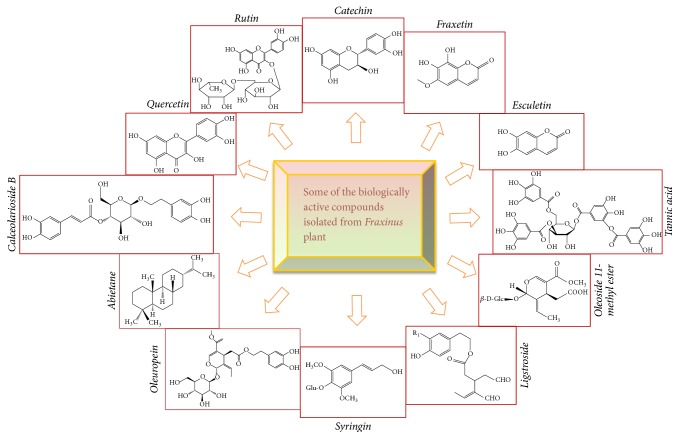
Some of the biologically active compounds isolated from* Fraxinus* plant.

*Fraxinus* plant has been accounted for its wide spectrum of biological activities including anticancer, anti-inflammatory, neuroprotective, antioxidant, anticytotoxic, antiaging, antimicrobial, and antihypertensive (Figure 1). Several* in vivo* and* in vitro* studies have been executed to describe its medicinal properties and to investigate the mechanism of action. Aggregated data have revealed a variety of bioactive medicinal components from different species of* Fraxinus* plant that exhibit various biological activities (Table 1).

### 2.1. Anticancer Activity

Phytochemicals obtained from medicinal plants, herbs, seeds, and fruits such as phenolic compounds (terpenoids, flavonoids, and carotenoids) have shown promising effects in suppressing proliferation and thus are anticarcinogenic [62]. Synthetic drugs have limited clinical utility as they are invariably associated with several toxic effects and drawbacks such as poor pharmacodynamic properties and nonselectivity [63]. Cell signaling pathways are the main pillars behind cell communication as they are essential for the regulation of cell proliferation and survival. Impairments in transduction pathways or cell signaling lead to various pathological conditions such as cancer [64]. Regulatory circuits and molecular machineries that govern cellular function and fate are mainly disrupted by oncogenic mutations, conferring tumor cells towards various traits that assist their malignant behavior. Various hallmarks underlie the establishment of tumor cells: substantiating angiogenesis, tissue invasion, disruption of apoptosis, limitless duplication potential, being unresponsive to antigrowth signals, metabolic reprogramming, and genomic imbalance [65]. Natural products are valuable anticancer lead structures for cancer drug discovery. Accumulated data by researchers commend that apoptosis induction in cancerous cells can result from various biological chemopreventive and chemotherapeutic agents [66–71].

**Figure 3:**
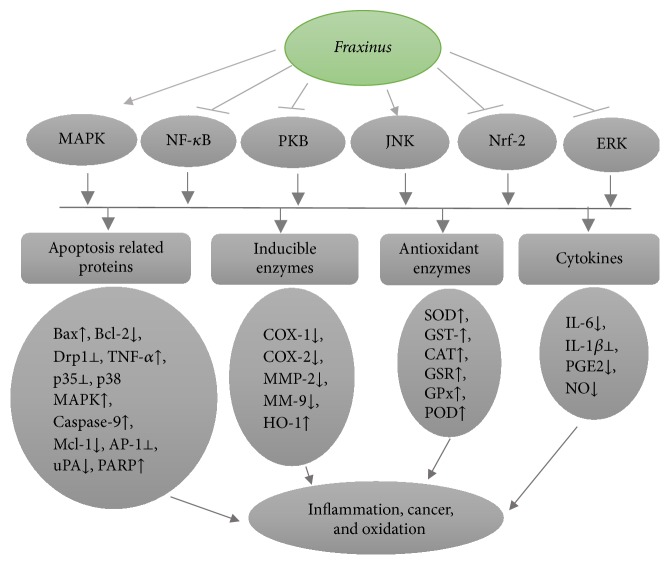
A diagram representing the molecular targets and mechanism of action for* Fraxinus* plant resulting in chemopreventive, anti-inflammatory, and antioxidant activity.* Fraxinus* plant and its isolated compounds cause effective inhibition of protein kinase B, nuclear factor-*κ*B, regulator of antioxidant metabolizing enzymes (Nrf-2), extracellular signal transducers (ERK), and modulation of mitogen activated protein kinases (MAPK) and c-Jun N-terminal kinase (JNK) followed by activity modulation of various apoptosis related factors, inducible enzymes, antioxidant enzymes, and cytokines which are associated with proliferation, inflammation, and other pathological conditions.

Various studies have revealed that* Fraxinus* plant is a notable biological drug candidate and has ability to inhibit carcinogenesis by targeting various signaling networks proteins associated with tumor cell multiplication (Figure 3). Here, we have reviewed anticancer activities of* Fraxinus* plant with an intent to provide a clear image to researchers about this medically important plant. Future efforts are required for the systematic identification of molecular targets for different compounds isolated from* Fraxinus* plant to enhance the possibilities of acquiring breakthrough vision in the field. Based upon previous researches, methanolic extract with bioactive components including polyphenols, flavonoids, and sterols from* Fraxinus micrantha* leads to induction of DNA fragmentation and production of NO thus conferring cells towards apoptosis in breast cancer cell line, MCF-7 [54]. Mistletoe extract obtained from host tree* Fraxinus* showed cytotoxicity against various cancerous cells including chronic myeloid leukemia K562 cells, human multiple myeloma RPMI-8226 cells, and murine leukemia L1210 cells via JNK-1/2, caspase-9, and p38 MAPK activation, Mcl-1 downregulation, and inhibition of PKB and ERK-1/2 phosphorylation. Abnobaviscum F extract specifically targets Mcl-1 at mRNA stage thus contributing to the activation of intrinsic caspase pathway. JNK-1/2 mediated caspase activation induced the proteolysis of PKB and PARP, which leads to cell death [56]. Stimulation of intrinsic caspase pathway and downregulation of cellular GSH in K562 lead to induction of apoptosis [56].* Fraxinus excelsior*, a novel herb, showed antiproliferative properties against numerous human cancerous cell lines: SKLC6 (lung carcinoma), AGS (Caucasian gastric adenocarcinoma), PLC/PRF/5 (liver hepatoma), SW742 (colorectal adenocarcinoma), A375 (melanoma cancer), and MCF-7 (breast ductal carcinoma) [55]. Glycosides isolated from the ethanolic extract of the bark of* Fraxinus sieboldiana* resulted in induction of apoptosis via activation of pro-caspase-8 in colorectal cancer HCT-8 cells [46]. Abietane extracted from* Fraxinus sieboldiana* exhibits cytotoxic activities against human ovarian A2780 cells and A549 (lung carcinoma) [57]. Further research work is mandatory to fill the gaps by mainly focusing on the molecular targets of these plant extracts in extrinsic and intrinsic mitochondrial apoptosis pathways. Data concerning many perspectives of the genus* Fraxinus* such as mechanism of action, phytochemistry (detail of bioactive components), and clinical trials employing scientific technologies is still very restricted which demand for supplementary studies peculiarly in humans. The details of all the compiled information concerning the effective inhibitory concentrations and molecular targets of bioactive components isolated from* Fraxinus* species are recorded in Table 2.

### 2.2. Anti-Inflammatory Activity

Even though inflammatory response varies among different diseases, principally diseased condition is linked with the production of prostaglandins which are formed from arachidonate by the action of cyclooxygenase (COX) isoenzymes. Anti-inflammatory drugs work by inhibition of enzymes COX-1 and COX-2 thus downregulating the production of prostaglandins. Proposed adverse effects of nonsteroidal type anti-inflammatory drugs (NSAIDs) such as renal and gastric toxicity provoke the need of alternatives with cyclooxygenases specific inhibitors [72]. Herbal medicines such as STW 1 (Phytodolor) with an active component obtained from* Fraxinus excelsior* are an acceptable substitute to COX-2-inhibitors such as rofecoxib and NSAIDs [73].

*Fraxinus* plant extract and its isolated compounds have a potential to modulate the activity of various key enzymes associated with inflammatory response (Figure 3). Oleuropein, phenolic compound isolated from the leaves of* Fraxinus rhynchophylla,* showed anti-inflammatory effects on BV-2 microglial cells via suppression of proinflammatory response by effective inhibition of Drp1-dependent mitochondrial fission [58]. Crude methanolic extract from the leaves of* Fraxinus xanthoxyloides* downregulated the production of inflammatory mediators and influx of leukocytes during* in vitro* and* in vivo* studies [27]. The 5-methoxyl aesculetin (MOA) from dried bark of* Fraxinus rhynchophylla* abrogates inflammatory response by its capability to block the activation of MAPK and activator protein-1 (AP-1) in RAW 264.7 cells. Much remains to be discovered about MOA as a propitious therapeutic agent for inflammatory diseases [59]. Screening of methanolic leave extracts from* Fraxinus floribunda* in rats suggests its significant pain relieving effects in inflammatory conditions [60]. Inhibition of myeloperoxidase (an enzyme released by triggered granulocytes and reported to produce harmful agent hypochlorous acid) by extracts from* Fraxinus excelsior* along with* Populus tremula* recommends its usage as anti-inflammatory drug [61]. Lipopolysaccharide (LPS) and interferon-gamma induced murine macrophage-like RAW 264.7 cells treatment by* Fraxinus rhynchophylla* extract showed an inhibition trend towards the amount of iNOS protein in dose-dependent manner suggesting its possible application as anti-inflammatory agent in autoimmune and chronic inflammatory diseases [74]. Inhibition of dihydrofolate reductase by aqueous ethanolic extract of* Fraxinus excelsior *is one of the possible mechanisms behind its anti-inflammatory activity [75]. Most of the available scientific literature has limited information about treatment duration, relevant doses, storage conditions, and controls for the assessment of bioefficacy of active components in plant extract arousing the need for further investigations. The detail of all the compiled knowledge about molecular targets of* Fraxinus* plant extracts is listed in Table 3.

### 2.3. Antioxidant Activity

In living systems free radicals as singlet oxygen (^1^O_2_), superoxide anion (O_2_^∙−^), hydroxyl radicals (OH^∙^), and other reactive oxygen species such as peroxynitrate, hydrogen peroxide (H_2_O_2_), OH, and hypochlorous acid are known to have damaging role and deleterious effects on cellular functions and paly outstanding role in several diseases [76]. Free radicals have capability to interact with cellular components and thus causing DNA damage, lipid peroxidation, and protein injuries [77]. Researchers have described a wide variety of plant extracts that have hepatoprotective activities usually associated with antioxidant activity as excessive production of free radicals overpowers the natural protective system causing hepatic damage [78–80].

Antioxidants are substances that have ability to reduce the harmful effects of these free radicals. Exogenous antioxidants primarily include natural as well as synthetic compounds having radical scavenging abilities. Hepatoprotective activities by* Fraxinus xanthoxyloides* leave extract against carbon tetrachloride (CCl_4_) induced oxidative stress in hepatic tissues in rats* (Rattus novergicus)* reduce the level of H_2_O_2_ and significantly increase the regenerative capacity of liver antioxidant enzymes (CAT, POD, SOD, GST, and GSR) (EC_50_ = 400 mg/kg) [76]. Oral administration of ethanolic extract from* Fraxinus rhynchophylla* [FR (EtOH)] against CCl_4_ induced hepatic fibrosis resulted in protective effects by its free radical scavenging ability against hepatocellular fibrosis in rats. FR (EtOH) has much action accordingly to dose: so 0.1, 0.5 g, and 1.0 g/kg body weight significantly upregulated the action of liver antioxidant enzymes (such as CAT, SOD, and GPx) and decreased the high activities of sGOT and sGPT. Furthermore, FR (EtOH) could cause inhibition of protein expression of uPA, TIMP-1, MMP-2, and MMP-9 in rats [81]. A novel compound fraxetin isolated from* Fraxinus rhynchophylla* showed dual antioxidative functions against metal and free radicals induced low density lipoprotein (LDL) oxidation. At low concentration (1–5 *μ*M) it has direct protective effects and at higher concentration fraxetin activated the nuclear factor Nrf-2/ARE, which were linked with the increased activity of glutathione S-transferase-alpha and HO-1. Fraxetin mediated induction of HO-1 has potential to enhance the detoxification of free radicals. [39]. Fraxetin showed protective activities against liver microsomal lipid peroxidation induced by Fe(+2) in rats with inhibitory rate of 60% at a concentration of 10(−6) mol × L(−1) [45]. Esculetin, a major component from* Fraxinus chinensis* extract (FCE), represented the strongest antioxidant activity against DPPH radicals associated with superoxide anions in xanthine oxidase system and esculetin also effectively inhibited the oxidation of 7′dichlorodihydrofluorescein diacetate (CM-H2DCFDA) and 5-(6-)dichloromethyl-2′ [48]. Esculetin (coumarin) isolated from* Fraxinus rhynchophylla* notably meliorated CCl_4_ induced hepatic toxicity by downregulating the aspartate aminotransferase and serum alanine aminotransferase and via upregulation of GSH-Px, SOD, and CAT and thus prevented the pathological condition associated with tissue injury [43]. Rutin isolated from* Fraxinus angustifolia* have notable radical scavenging activity via inhibition of ethane release from Fenton-type oxidants induced 1-keto-4-methylthiobutyrate (KMB) and inhibition of nitrogen monoxide (NO) release from hydroxylamine [82]. Mechanism of action of* Fraxinus* plant is mainly associated with the upregulation of various antioxidant enzymes (Figure 3).

### 2.4. Antimicrobial Activity

Scientists have demonstrated a variety of chemical compounds from plants, notably the secondary phytochemicals known to exhibit antimicrobial activity and which are effective against multidrug resistance microorganisms. The concern of resistance provoked the need of effective and eco-friendly alternatives [83]. Treatment of bacterial cultures (*Staphylococcus aureus*,* Pseudomonas aeruginosa*,* Streptococcus lactis*,* Escherichia coli,* and* Mycobacterium phlei*) with plant extracts using microplate resazurin assay for screening showed that* Fraxinus pennsylvanicca* exhibits notable antibacterial activities with MICs ranging from 62.5 to 1000 *μ*g/ml [84].

Fraxetin (one of the main constituents of* Fraxinus rhynchophylla*) inhibitory effects against* Staphylococcus aureus* proliferation were investigated by treatment of bacterial cultures with fraxetin at a concentration of 0.05 mg/ml. The mechanisms associated with antimicrobial action of fraxetin indicated the highest inhibition of topoisomerase-1 and topoisomerase 2 and remarkable increase in membrane permeability. Additionally, macromolecules such as DNA, RNA, and proteins are decreased to 33.86, 48.96, and 55.74% [85]. Further studies are required with more microbial species to assemble data related to* Fraxinus* plant that can act as potential antimicrobial agent.

### 2.5. Neuroprotective Activity

Neuroprotective mechanism for natural compounds depends upon their free radical scavenging ability generated by neurotoxin and oxidative induced processes in nerve cells [86]. Glutamate as the abundant mammalian neurotransmitter and excessive extracellular level of glutamate cause the activation of glucose receptors and overloading of neuronal calcium (Ca^+2^) level that leads to glutamate induced neuronal injury [87, 88]. Oleuropein isolated from* Fraxinus rhynchophylla* can be used as neuroprotective agents against colchicine induced neurodegenerative diseases as it significantly recovered memory and learning retention [89]. An attempt to elucidate the mechanism of action of* Fraxinus* plant extract is that it increases the Bcl-2 expression, inhibits the translocation of mitochondrial apoptosis-inducing factor (AIF) to the cytoplasm, and decreases the Bax expression. Furthermore, it causes the regulation of phosphorylation of Drp1 at serine 637 and reduction in the number of cells with fragmented mitochondria [42].

*F. rhynchophylla* have remarkable inhibitory activity against neuronal cell damage induced by Abeta (25–35) by downregulating the activity and expression of caspase-3, reducing the cleaved PARP and DNA fragmentation with an effective concentration of 20 *μ*M [90]. Moreover esculin isolated from* Fraxinus sieboldiana* blume has neuroprotective properties on cytotoxicity stimulated by dopamine in human neuroblastoma SH-SY5Y cell line via downregulating the GSH levels, upregulating the SOD activity, inhibiting the apoptosis-inducing factor (AIF), the discharge of cytochrome c and the expression of activated caspase 3, and regulating the Bax, Bcl-2, and p53 proteins [91]. Most of the studies focus on few chemical constituents and data about the pharmacokinetics of the whole plant extract is limited.

### 2.6. Antifungal Activity

Protective products originated from natural substances mainly from plants as alternative to synthetic fungicides is the spotlight issue nowadays [92]. Natural antimicrobial products are inexpensive and they have a potential for implementation in fungal pathogenic systems [93]. The antifungal activity against* Schizosaccharomyces octosporus* and* Candida albicans* cultures with inhibitory concentration value ranging from 62.5 to 1000 *μ*g/mL shows that* Fraxinus pennsylvanicca* plant extract exhibits notable antifungal activity [84].

### 2.7. Antihypertensive

Researchers have described a variety of bioactive compounds found in natural substances that play vital roles in prevention and treatment of cardiovascular diseases caused due to vasodilation and hypertension [94]. Administration of* Fraxinus excelsior *L. seed extract (FESE) orally at a daily dose of 20 mg/kg or 40 mg/kg body weight of animal in spontaneously hypertensive rats improved acetylcholine relaxation in aorta, decreased plasma and liver malondialdehyde levels, and increased plasma antioxidant capacity [95]. Protective hypotensive effects of* Fraxinus excelsior* extract were evaluated in spontaneously hypertensive and normotensive rats and oral administration of 20 mg/kg/day for about 3 weeks resulted in significant decrease in systolic blood pressure (SBP) [96]. Nuzhenide and GI3, the novel compounds from* Fraxinus excelsior *L. seed extract (FXE), decrease the systolic blood pressure significantly in spontaneously hypertensive rats (SHR) and obese Zucker rats at a dose concentration of 200 mg/kg [32].

### 2.8. Antimalarial

An alarming situation of resistance against antimalarial drugs leads to the requirement of affordable treatment from medicinal plants [97]. Scientists have described antiplasmodium potential from variety of plant extracts [98].* In vitro* studies to check the effects of lipo and hydrophilic extract from the bark of* Fraxinus excelsior* on the growth of asexual stages of* Plasmodium falciparum* suggest that it has significant inhibitory effects on the development of asexual stages of* Plasmodium falciparum* [99].

### 2.9. Antitoxoplasmosis

Toxoplasmosis, an intracellular parasitic protozoan infection caused by* Toxoplasma gondii,* is usually asymptomatic but has serious clinical manifestations in immunocompromised individuals [100]. Herbal extracts that have important role in the regulation of immunity can serve as effective and secure medicine against toxoplasmosis [101]. Serious toxic effects associated with the application of antitoxoplasmosis drugs like sulfadiazine and pyrimethamine arouse the urgent need of safe and effective alternatives.* In vitro* evaluations of antitoxoplasma activity of oleuropein and its metabolites isolated from* Fraxinus rhynchophylla* showed a good efficacy and higher selectivity as an anti-*T. gondii* compound. (EC_50_ = 139 mg/ml) [41]. However, the results are not sufficient and should be approved by related tests along with clinical trials. The culpable compound should be subjected to isolation from extract and purified for further inquiry.

### 2.10. Antiaging

Most of the plants that have high percentage of polyphenolic compounds have been widely used in cosmetics for their antiaging properties [102]. Esculetin from* Fraxinus chinensis* extract resulted in protective effects against photoaging via downregulating the MMP-1 mRNA in a dose-dependent fashion due to its free radical scavenging nature [48].

## 3. Other Biological Activities

Some other biological activities of medically significant compounds isolated from* Fraxinus* species are as follows: diterpenes analogs from* Fraxinus sieboldiana* resulted in a prohibitory activity opposite to H5N1 avian influenza virus. Inhibitory concentration (IC_50_) reported to be 4.8 *μ*M [57]. Evaluation of methanolic extracts from* Fraxinus floribunda* in rats at a dose concentration of 400 mg/kg/oral reveals its significant antinociceptive properties and its potential for relieving pain in pathological conditions such as inflammation [60]. Polyphenolic compounds as catechin, rutin, quercetin, and tannic acid from* Fraxinus angustifolia* bark and leaf extracts incorporated with different nanovesicles to increase the skin bioavailability found to be effective for their wound healing potential which is associated with their antioxidant and anti-inflammatory activity [31].* Fraxinus excelsior* bark extract for its free radical scavenging activities and for tyrosinase elastase and collagenase prohibitory activities suggest its use in dermocosmetic industry [103]. Glucevia, an effective* Fraxinus excelsior* herbal extract that competently balances fructosamine and blood glucose concentrations, significantly augmented adiponectin-leptin ratio in obese and overweight older rats via oral administration of Glucevia immediately after sugar rich meals [104, 105].* In vitro* assessment of hydroxyframoside B isolated from ethanolic extract of stem bark of* Fraxinus rhynchophylla* provided an approach to decrease obesity via downregulating the pancreatic lipase activity thus restricting the absorption of lipid by pancreas [36]. Treatment of streptozotocin- (STZ-) induced diabetic rats by* Fraxinus angustifolia *leaf extract (25 and 50 mg/kg) resulting in considerable hypoglycemic effects with significant reduction in malondialdehyde levels in short interval of time providing a way for the treatment of diabetes [106]. Oral administration of hydroethanolic extracts from* Fraxinus ornus* at dose concentrations of 10 or 50 mg/kg body weight in nicotinamide-streptozotocin-induced diabetic mice showed potent antihyperglycemic activity. Identification and isolation of lead structures from hydroethanolic extract for novel antidiabetic drug development are commended [107].* Fraxini cortex*, a traditional Chinese medicinal plant, possesses significant antidiarrheal properties having a notable effect on Cl(−) transport as simply the diarrhea is altered movement of Na^+^ and Cl^−^ ions [108]. Drugs that work to decrease the elevated blood pressure in body by increasing the amount of urine and urinary sodium excretion are known as diuretics. A study on herbal medicines has revealed that* Fraxinus excelsior* extract promotes diuresis and thus potentially can be used as hypotensive agent [109].

## 4. Conclusions and Future Perspectives

This review reveals that* Fraxinus* plant is a valuable drug candidate with its potential anticarcinogenic, anti-inflammatory, antioxidative, and neuroprotective properties. Various* in vitro* and* in vivo* studies results have demonstrated its several applications in biological systems. Stem bark, root bark, and leave extract of this plant have wide applications in traditional folk medicines since ancient times.* Fraxinus* plant derivative analogs along with pharmacodynamics and pharmacokinetics may also strengthen future advances.* Fraxinus* plant extracts can serve as template for new drug design and synthesis of new compounds for various human diseases treatments. To date, most of the researchers do not figure out the chemical ingredients of the plant extracts. Then, various pharmacological perspectives of* Fraxinus* plant such as proper dosage and clinical effectiveness are yet to be elucidated. There is a need to identify the toxicological limits for certain organs like liver and kidney. The molecular mechanism and exact protein targets of potent bioactive molecules from* Fraxinus* plant also deserve to be further investigated. With regard to ongoing investigations on* Fraxinus* plant's biological applications further scientific experimentations and safety profiling are required to make understandings more clear and obvious in the treatment and prevention of various diseases.

## Figures and Tables

**Table 1:** Compounds isolated from various species of *Fraxinus* plant and their biological activities.

Plant species	Isolated compounds	Parts used	Biological activities	References
*Fraxinus angustifolia*	Tannic acid, catechin quercetin, rutin	Bark, leaves	Antioxidant, anti-inflammatory, wound healing potential	[31]

*Fraxinus excelsior*	Nuzhenide, GI3, GI5, ligstroside, oleoside 11-methyl ester, 1′′′-O-beta-D-glucosylformoside, excelsides A, excelsides B, oleoside dimethyl ester, coumarins	Seeds	Antihypertensive, antihypertriglyceridemia, adipocyte differentiation inhibitory activity, antidiabetic, antihyperglycaemic, anti-inflammatory, antirheumatic	[32–35]

*Fraxinus rhynchophylla* C	Hydroxyframoside B 2′′-hydroxyoleuropein, oleuropein, ligstroside, syringin, esculin, fraxetin, fraxetin-8-O-[11′-methyl-oleosidyl-(7′→ 6′′)]-beta-D-glucopyranoside, esculetin	Stem bark, dried stem bark, root bark	Pancreatic lipase inhibitory activity, inhibitor of adipocyte differentiation in 3T3-L1 cells, neuroprotective (protection against Abeta (25–35)-induced neuronal toxicity), antidyslipidemia, renoprotective, metal and free radical induced LDL oxidation inhibitor, antiatherosclerosis, antioxidant, PTP1B inhibitor, antitoxoplasmosis, glutamate-induced neuronal HT-22 cell death inhibition, hepatoprotective	[36–43]

*Fraxinus griffithii*	7-epi-7-O-(E)-caffeoylloganic acid, griffithosides C	Leaves	Antioxidative	[44]

*Fraxinus sieboldiana*	Abietane, C20-norabietane, 6, 7-di-O-beta-D-glucopyranosylesculetin, aromatic glycosides, plantasioside, Calceolarioside B, Esculetin	Stem bark	Anticarcinogenic {cytotoxic activities against A549 and A2780 {IC_50_ = 6.0 *μ*M, 1.7 *μ*M}}, protective against H5N1 avian influenza virus, inhibition against the discharge of *β*-glucuronidase in platelet-activating factor induced polymorphonuclear rat leukocytes, antioxidative, TNF-*α* secretion inhibitor (IC50 = 1.6 *μ*M), inhibition against liver microsomal lipid peroxidation induced by Fe(+2)-cystine, anticancer against human colon cancer cell line (HCT-8) with IC_50_ = 3.4 *μ*M, HIV inhibitor (IC_50_ = 0.1 mg/ml and 0.5 mg/ml)	[45–47]

*Fraxinus chinensis*	Esculin, esculetin, chinensisol	Twig skin, trunk bark	Antioxidant against DPPH radicals, antiphotoaging, quinone reductase (QR) inducing activity	[48, 49]

*Fraxinus mandshurica*	Calceolarioside A	Leaves	Immunosuppressant (inhibition of IL-2 and IgE production in mouse spleen cells and U266 cells)	[50]

*Fraxinus dimorpha*	(E)-Nerolidol, eugenol	Leaves	Antioxidant, antifungal (MIC = 1.8–3.8 mg/mL)	[51]

*Fraxinus ornus*	Esculin	Stem bark	Anti-inflammatory against zymosan- and carrageenan-induced paw oedema	[52]

*Fraxinus bungeana*	Esculetin, fraxetin		Analgesic, anti-inflammatory (superoxide scavenging effects on the xanthine-xanthine oxidase-cytochrome c system)	[53]

**Table 2:** Molecular targets of *Fraxinus* plant in various cancer types.

Type of cancer	Cell lines	EC50/conc.	Molecular targets	References
Breast	MCF-7	18.95 *µ*g/ml, 35.622 *µ*g/ml	NO↑	[54, 55]
Leukemia	K562, RPMI-8226, L1210	20 *μ*g/ml	PKB phosphorylation⊥, Mcl-1↓, ERK-1/2⊥	[56]
Gastric	AGS	37.338 *μ*g/ml		[55]
Colon	SW742, HCT-8	31.092 *μ*g/ml, 3.4 *μ*M		[46, 55]
Lung	SKLC6, A549	68.072 *μ*g/ml, 6.0 *µ*M		[55, 57]
Skin	A375	51.849 *μ*g/ml		[55]
Ovary	A2780	1.7 *µ*M		[57]
Liver	PLC/PRF/5	21.036 *μ*g/ml		[55]

**Table 3:** Anti-inflammatory properties of *Fraxinus* plant against various disease models and its molecular targets.

Assay	Organism tested	Dose/conc.	Molecular targets	References
Anti-inflammatory effect of oleuropein on LPS-induced BV-2 microglial cells	*In vitro* (BV-2 murine microglial cells)		ERK⊥, NF-*κ*B⊥, LPS-induced Drp1 dephosphorylation⊥, ROS⊥	[58]

Protective effects of methanolic extract from *F. xanthoxyloides* leaves on inflammatory mediators	*In vitro* (LPS-activated RAW 264.7 cells, TNF-*α* activated NF-kB in 293/NF-kB-Luc HEK cells), *in vivo *(rat)	5.98 *μ*g/ml (Luc HEK cells), 6.59 *μ*g/ml (RAW 264.7 cells), 200 mg/kg *(in vivo)*	TNF-*α* induced production of NF-*κ*B⊥, NO↓ *(in vitro)*, IL-6↓, PGE2↓, TNF-*α*↓, LPS-instigated NO⊥ *(in vivo)*	[27]

Modulation of cytokine expression by 5-methoxyl aesculetin (MOA) in LPS-stimulated RAW264.7 macrophages	*In vitro *(RAW 264.7 cells)	25.32 *μ*g/mL	TNF-*α*⊥, interleukin-6⊥, interleukin-1*β*⊥, ERK1/2⊥, p38 MAPK⊥, AP-1⊥, PGE_2_⊥, cyclooxygenase-2↓, TNF-*α* mRNA↓, nitric oxide (NO) synthase↓	[59]

Protective activities of methanolic extract of *Fraxinus floribunda* Wallich against inflammation	Wistar Albino rats, Swiss Albino mice	400 mg/kg/p.o.		[60]

Anti-inflammatory effects of *Fraxinus excelsior* during *in vivo* and *in vitro* studies	*In vitro, in vivo*		T-cell activation⊥, arachidonic acid cascade⊥	[61]
